# A Case of Diffuse B-Cell Lymphoma Presenting as a Rash on the Chest and Abdomen

**DOI:** 10.7759/cureus.79751

**Published:** 2025-02-27

**Authors:** Saurabh Dubey, Ashraf Sliem, Mohamed Akil, Nasir Gondal, Anjali Bakshi

**Affiliations:** 1 Internal Medicine, Flushing Hospital Medical Center, New York City, USA; 2 Hematology-Oncology, Flushing Hospital Medical Center, New York City, USA; 3 Infectious Disease, Flushing Hospital Medical Center, New York City, USA

**Keywords:** confounding diagnoses, drug rash, maculo-papular rash, primary cutaneous diffuse large b-cell lymphoma, rash on chest and abdomen

## Abstract

Diffuse large B-cell lymphoma (DLBCL) can present with skin manifestations either as a primary cutaneous DLBCL or as systemic DLBCL with cutaneous manifestations. The majority of cutaneous cases present with erythematous nodules on the legs, most commonly the lower legs. DLBCL presenting at a cutaneous site can be mistaken for cellulitis, a chronic venous ulcer, or psoriasis. Here, we present a case of DLBCL manifesting as a rash on the upper chest, shoulder, and abdomen. The case is notable in the fact that the rash developed while the patient was hospitalized and being treated for sepsis, with a drug-induced rash being a confounding diagnosis.

## Introduction

Diffuse large B-cell lymphoma (DLBCL) is a malignant neoplasm arising from mature B cells. DLBCL accounts for 28% of all lymphoid neoplasms, with 27,000 cases occurring in the United States every year [1].

Patients typically present with rapidly expanding nodal masses; however, approximately 40% of cases present with extranodal involvement, of which approximately half present with Stage 1 disease [1]. Primary cutaneous B-cell lymphomas are defined as malignant B-cell proliferations presenting with cutaneous involvement alone and no evidence of extracutaneous manifestations when complete staging has been performed [1].

The most common extranodal site of DLBCL is the gastrointestinal tract, with the skin being the second most common [1-3]. Skin involvement at presentation can be in the form of plaques, papules, nodules, or ulcers [4]. Cases of DLBCL presenting on sites such as the trunk, shoulder, neck, or scalp are rare, and few have been described in the literature [3-5].

## Case presentation

**Figure 1:**
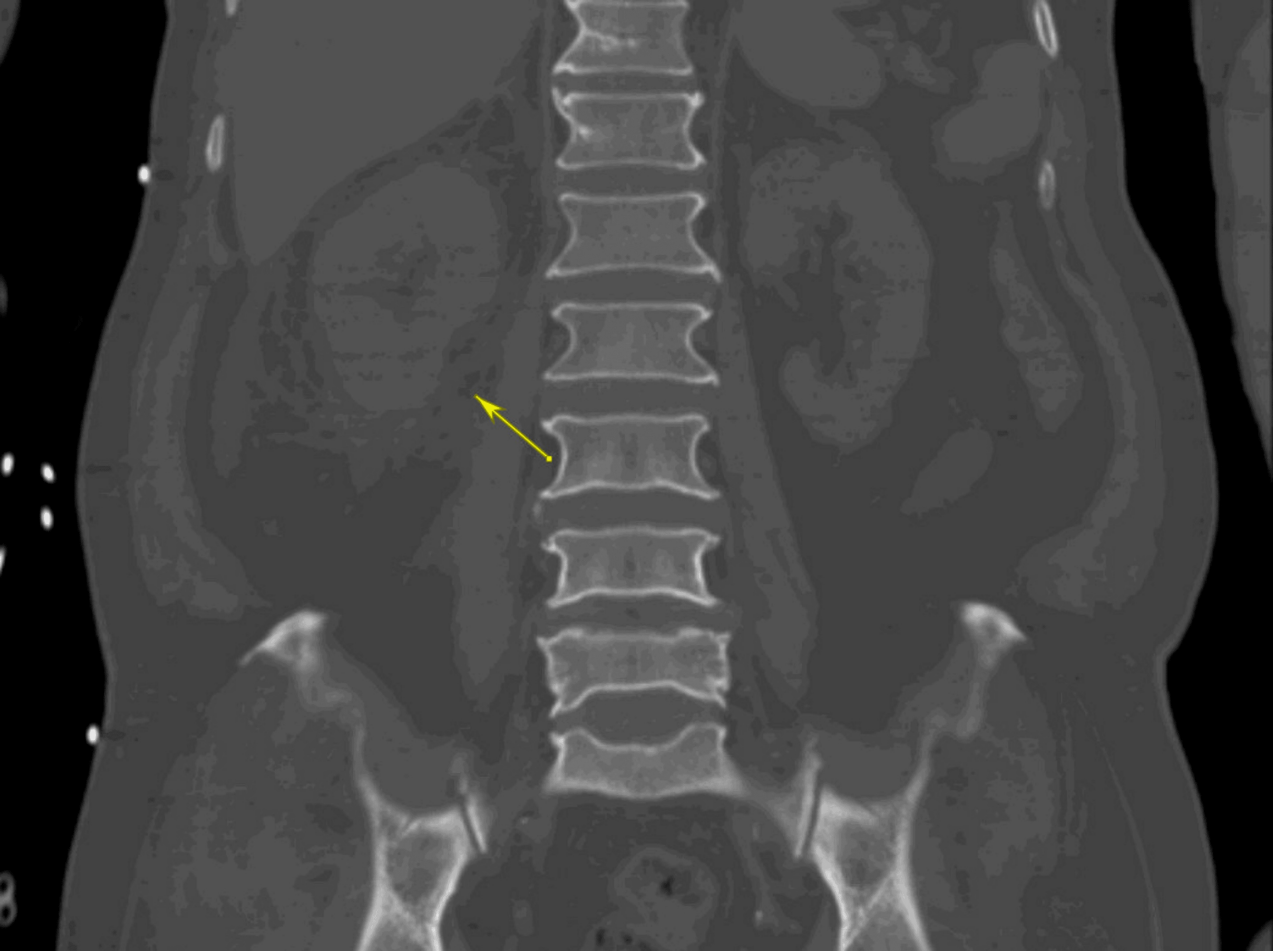
Coronal section of patient's CT scan of the abdomen and pelvis showing fat stranding around the right kidney (yellow arrow)

The patient was a 72-year-old male of South Asian descent who presented from home with respiratory distress and hypotension to the emergency room (ER) of Flushing Hospital Medical Center. The patient had to be intubated in the ER due to respiratory distress and was then admitted to the Medical Intensive Care Unit. Investigative workup was also remarkable for a computed axial tomography (CAT) scan of the abdomen without contrast that showed perinephric inflammation near the right kidney suggestive of pyelonephritis (Figure 1), and he was treated with cefepime empirically for sepsis thought to be secondary to pyelonephritis. The diagnosis of sepsis was made as the patient met systemic inflammatory response syndrome (SIRS) criteria with an elevated leukocyte count of 22,000 and a temperature of 102.1°F on admission. Blood cultures grew extended-spectrum beta-lactamase-producing (ESBL) *Escherichia coli*, so the patient was switched from cefepime to meropenem. A CAT scan of the chest was also done, which showed shotty mediastinal lymphadenopathy but did not show any masses or otherwise clinically significant findings. The patient later had a drop in his hemoglobin, for which a CT angiogram of the abdomen and pelvis was done to rule out gastrointestinal bleeding, which was significant only for urinary bladder wall thickening suggestive of cystitis. This scan did not show any signs of bleeding or any masses or lymph nodes suspicious of malignancy. 

**Figure 2:**
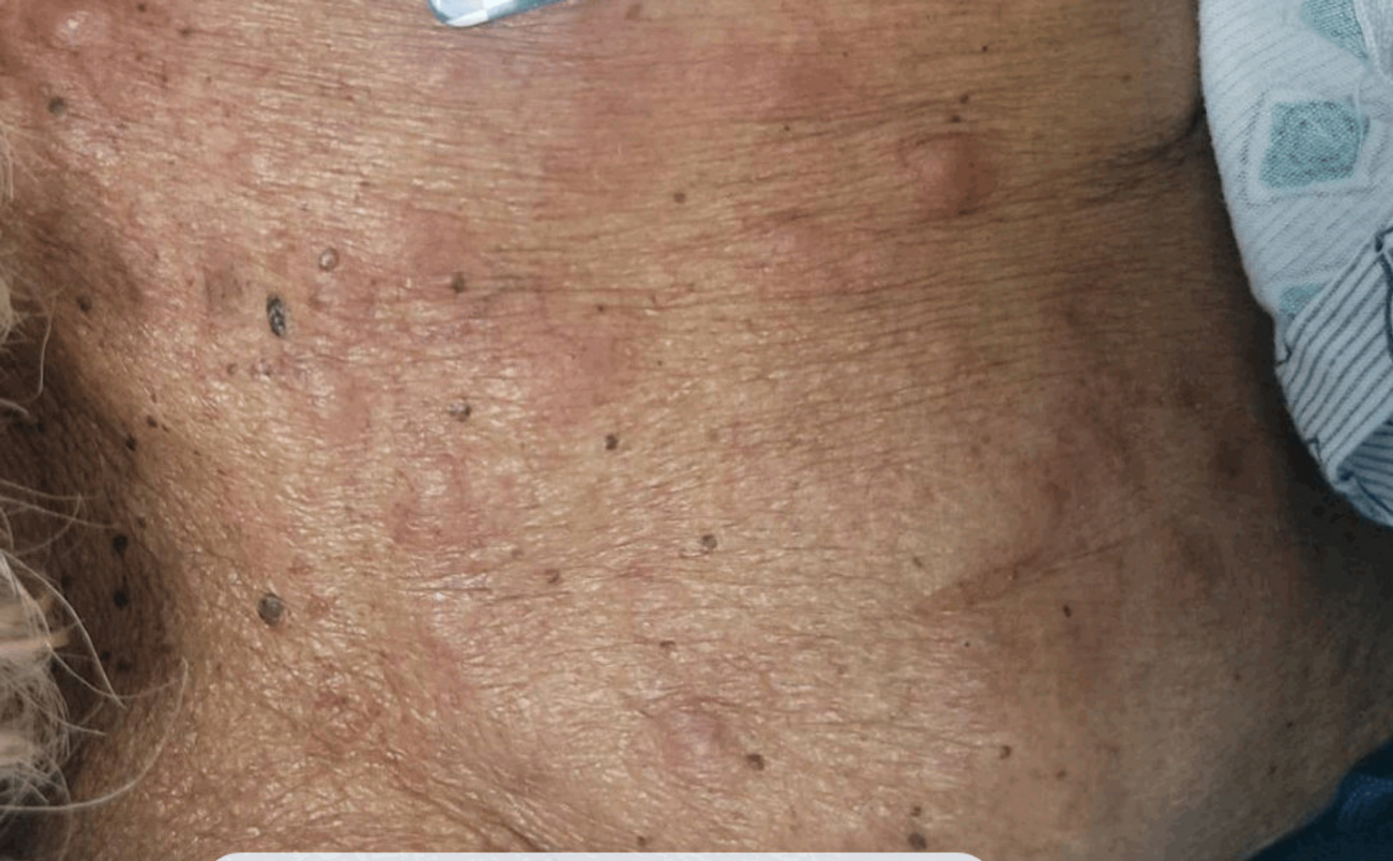
Rash (upper right chest)

Ten days after he initially presented to the ER, the patient began to develop erythematous nodular skin lesions on the upper chest, shoulder, and abdomen. At the time, the most likely cause of the skin lesions was thought to be an atypical drug reaction. A photograph of the rash is included in Figure 2.

However, because of the characteristics of the rash, which was atypical for a drug reaction, a skin punch biopsy was done to confirm the diagnosis. By this time, the patient had considerably improved and was discharged to follow-up on an outpatient basis regarding the results of the skin biopsy.

**Figure 3:**
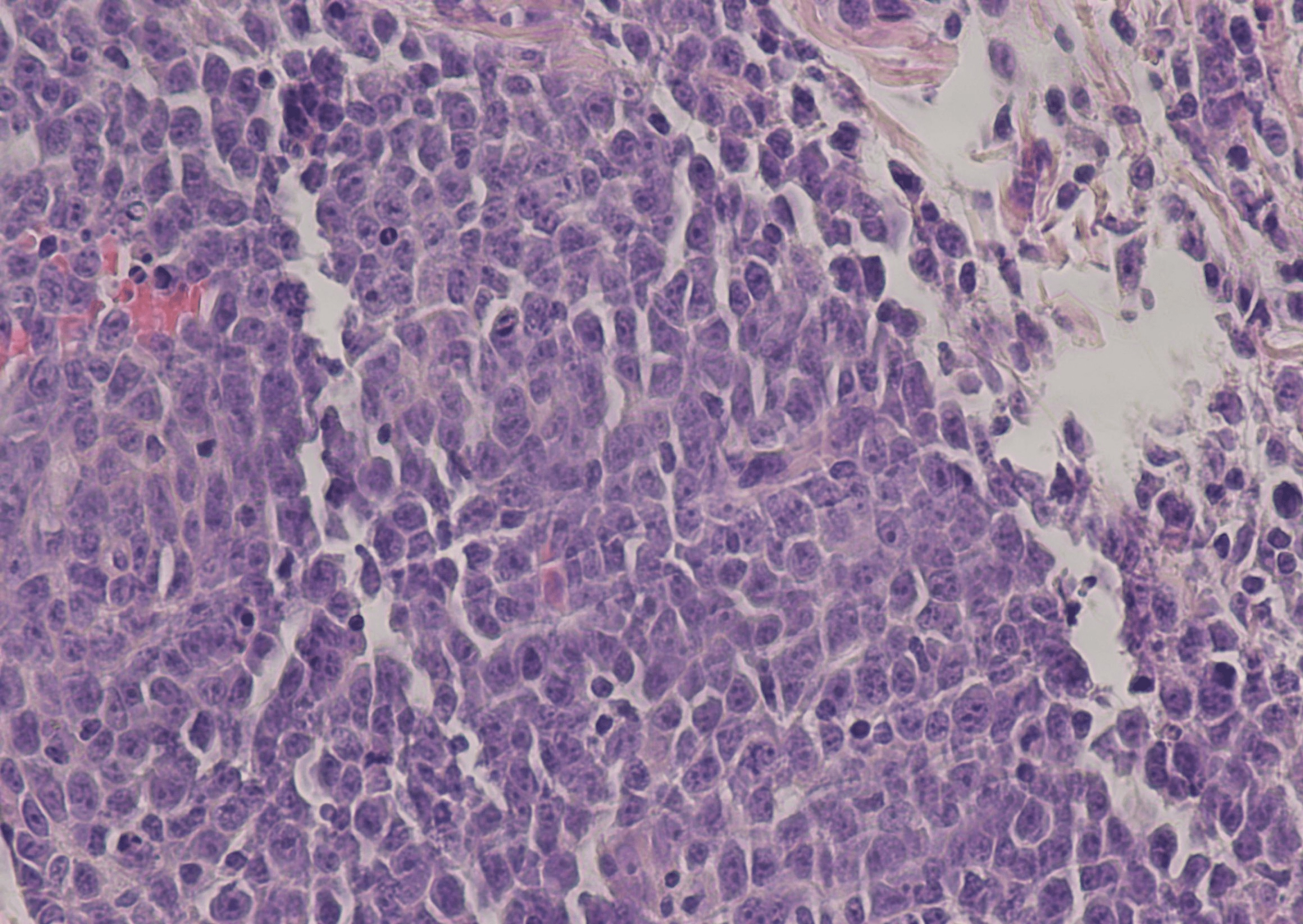
Histopathology of the skin punch biopsy from the patient's chest, H&E stain, showing highly atypical large cell infiltrate, with lymphocytes having a blastic appearance H&E: Hematoxylin and eosin

Histology of the skin punch biopsy was done and showed a skin specimen with highly atypical large cell infiltrate, with lymphocytes having a blastic appearance in the 15- to 20-micron range (Figure 3). The lymphocytic cells show scant basophilic cytoplasm, increased nucleus/cytoplasmic ratio, open chromatin, and prominent nucleoli. Large cells with immunoblastic features predominating over centroblasts were seen. Immunohistochemistry was done and was read as showing lymphomatous cells positive for CD20, MUM1, Ki-67, CD43, and BCL2. Interpretation based on histology and immunohistochemistry was determined as diffuse large B-cell lymphoma, immunoblastic (ABC-like). Fluorescence in situ hybridization (FISH) hybridization was done, which showed no evidence of myelocytomatosis oncogene (MYC) rearrangement, BCL2-IGH gene rearrangement, or BCL6 breakpoint translocation. The patient passed away before further workup or treatment could be done.

## Discussion

Cutaneous manifestations of diffuse large B-cell lymphomas are rare, and they are initially frequently confused with other possible etiologies, including cellulitis or venous stasis ulcers. Our patient presented with a nodular erythematous rash, which is not typical for DLBCL.

Initially, the rash was suspected to be possibly drug-induced or infectious in etiology, given it was closely associated with antibiotic use and sepsis. However, the erythematous nodular nature of the rash was not consistent with a typical drug-induced rash, so further workup via skin biopsy was done, revealing the diagnosis. Differentials other than atypical drug rash at the time included atypical bug bites and cutaneous pseudolymphoma.

Computed tomography (CT) head, CT angiogram of the abdomen, and CT chest without contrast showed no signs of systemic disease or alternative primary lesion; as such, the diagnosis of primary cutaneous DBCL was made in this case.

## Conclusions

Our patient presented with a rash that was initially thought to be a side effect of antibiotic therapy (the patient had very recently received cefepime and meropenem), and because the patient had no primary lesions seen on radiological imaging prior to this, the possibility of the rash being due to a lymphoma was not considered at the time. However, because the features of the rash were not typical of that of a usual drug-induced rash, the decision was made to request a skin biopsy. This case demonstrates the need to keep a broad differential in patients who present with atypical rash and the importance of histological diagnosis via skin biopsy in confirming the diagnosis.

## References

[1] Morton LM, Wang SS, Devesa SS (2006). Lymphoma incidence patterns by WHO subtype in the United States, 1992-2001. Blood.

[2] Alaibac M, Bordignon M, Pennelli N Primary subcutaneous B-cell lymphoma: case report and literature review. Acta Derm Venereol.

[3] Møller MB, Pedersen NT, Christensen BE (2004). Diffuse large B-cell lymphoma: clinical implications of extranodal versus nodal presentation--a population-based study of 1575 cases. Br J Haematol.

[4] Kilaru S, Panda SS, Mishra S (2021). Cutaneous involvement in diffuse large B cell lymphoma at presentation: report of two rare cases and literature review. J Egypt Natl Canc Inst.

[5] Mishra S, Shelly D, Vinu BKV, Bharadwaj R (2017). Primary cutaneous B-cell lymphomas: case report of two cases. Indian J Dermatol.

